# Supplementary material for: Patterns of Intron Gain and Loss in Fungi
Source: PLoS Biol. 2004 Nov 30;2(12):e422. doi: 10.1371/journal.pbio.0020422 (PMC532390; doi:10.1371/journal.pbio.0020422)
Supplement: Table S1 — Also available at http://genes.mit.edu/NielsenEtAl/. (4.3 MB ZIP). [file pbio.0020422.st001.zip › NielsenEtAl/html/168.html]

AN1019.1.NCU05204.1.MG07145.1.FG09616.1


```
 CLUSTAL W (1.82) Multiple Sequence Alignments - Introns Inserted


Sequence 1: NCU05204.1	769 aa
Sequence 2: MG07145.1	766 aa
Sequence 3: FG09616.1	733 aa
Sequence 4: AN1019.1	751 aa
Alignment Length: 781 aa
Number Identitical Residues: 395 aa
Alignment Score (without introns) 19155


MG07145.1 	MAPASRAPLGVG---SGRDDIEVT2WNYLQNGITRIMNNLQDGMTMET0YMGIYT2SVHN
NCU05204.1	MPPATMMMGAYGQQPSGSDGIDST2IN-------KIMTNLQEGLDMSS0YMGIYT2AVHN
FG09616.1 	MAAKMGAPSQMPP-IPNREDIGAT2WTYLQAGIARVMNDLEQ------~------~----
AN1019.1  	MAPLMPQA-------PHKDDLEET2WVFLENGINNVMVKLEDGVDMKN0YMALYT2AVHN
          	*..            .  :.:  *     . .  .:* .*::.   ..   .  : :  .

MG07145.1 	FCTSQKALGFSNSPG---PPAAAHRGA1HLLGEDLYKKLMKYLSDHLEDLLVQSKAHTGE
NCU05204.1	FCTSQKANGGSSSPSNHLPGIGAQRGA1HLLGEDLYKKLANYLTDHLKHLVSEAEAHKDE
FG09616.1 	--------------------------A1HLLGEELYNKLIDYLKHHLEDLVNQSKTHTDE
AN1019.1  	FCTSHKAVSG--------QAIQAQRGA1HLLGEELYRKLGEYLSRHLEWVHGESMGHTDE
          	 .:: .: .             :  .* *****:**.** .**. **: :  ::  *..*

MG07145.1 	ALLSFYIREWDR~YTTAAKYIHHLFRYLNRHWVKREIDEGKKHIFDVYTLHLVQWKNVLF
NCU05204.1	ALLAFYIREWQR~YTNAAKYIHHLFKYLNRHWVKREMDEGKKNIYDVYTLHLVQWRDVLF
FG09616.1 	ALLAFYIKEWGR~YTVAAKYIHHLFRYLNRHWVKREIDEGKKNIYDVYTLHLVQWRKELF
AN1019.1  	ALLSFYIREWQR2-------------YLNRHWVKREIDEGKKNVYDVYTQHLVKWKEDFF
          	***:***:** *              **********:*****:::**** ***:*:. :*

MG07145.1 	DKVHEKVMDAVLALVERQRNGETIEYGQIKQILDSMVSLGLDENDASKTTLDCYRFHFEK
NCU05204.1	QAVSKKVMDAVLKLVERQRLGETIEYTQIKQVVDSFVSLGMDEGDNTKTTLEVYRYHFER
FG09616.1 	EKVHAKVMDAVLKLVEKQRNGETIEHNQIKQVVASFVSLGLDEADPNKSTLDVYRYNFER
AN1019.1  	LKVHEKVMGAVLKLVEKQRNGETIEQSRIKSIVDSFVSLGLDETDPTKSTLEIYRYYFQR
          	  *  ***.*** ***:** *****  :**.:: *:****:** * .*:**: **: *::

MG07145.1 	PFIAATEAYYQKESRAFVAENTVVEYMKKAEARLEEEEERVRMYLHPDIAVALKRCCNTA
NCU05204.1	PFLEATKVFYQNESKQFVAENSVVEYMKKAEARLAEEEERVRMYLHPDIALHLKRTCNQA
FG09616.1 	PFLGATKEFYVAESKQFVAENSVVEYMKKAETRLAEEEERVRMYLHGDIALPLKKTCNQA
AN1019.1  	PFLEATRIYYEDESRQFVADNSVVEYMKKAEIRLEEEKARVGLYLNNDISKDLTSTCLDV
          	**: **. :*  **: ***:*:********* ** **: ** :**: **:  *.  *  .

MG07145.1 	LIADHSAILRDEFQVLLDNDREDDMARMYNLLARISDGLDPLRQKFEAHVRQAGLTAVAK
NCU05204.1	LIAEHSTLLREEFQALLDNNREDDMRRMYSLLSRIPDGLEPLRTRFEAHVRKAGLAAVAK
FG09616.1 	LIADHSPLLREEFQVLLDNDREDDMARMYNLLSRIPDGLDPLRARFETHVRKAGLAAVQK
AN1019.1  	LVTAHSELLRDEFQPLLDNERQDDLARMYRLLSRIKDGLDPLRTKFEAHVRRAGLSAVEK
          	*:: ** :**:*** ****:*:**: *** **:** ***:*** :**:***:***:** *

MG07145.1 	VASGQGEKLEPKVYVDALLEVHTQYQGLVKRAFNDEPEFTRSLDNACREFVNRNDVCKSG
NCU05204.1	VAAD-ADKLEPKVYVDALLEIHTQYQGLVERAFNKEPDFTRSLDNACKEFVNRNEVCKSG
FG09616.1 	IQSAEGDKLEPKVYVDALLDIHTQYQGLVERAFKNEPEFTRSLDNACREFVNRNEVCKSG
AN1019.1  	VAAE-GDSFEPKMYVDALLSVHTRYHSLVKEAFNGESEFVRSLDNACREFVNRNKICKSG
          	: :  .:.:***:******.:**:*:.**:.**: *.:*.*******:******.:****

MG07145.1 	SNKSPELLAKYTDVLLRKSGSAVEEEELENTLSQIMTVFKYIEDKDVFQKFYSRMLARRL
NCU05204.1	SNKSPELLAKYTDVLLRKSSTGVEEAELENTLTQIMTVFKYIQDKDVFQKFYSRMLARRL
FG09616.1 	SSKSPELLAKYADVLLRKSSTSIEESELEHTLNQIMTVFKYIEDKDVFQKFYSRMLARRL
AN1019.1  	STKTPELLAKYTDSLLKRGSKAAEESELEEMLVQIMTVFKYIEDKDVFQKFYSKMLAKRL
          	*.*:*******:* **::.... ** ***. * *********:**********:***:**

MG07145.1 	VHTNSSSDDAETSMISKLKEACGFEYTNKLQRMFQDMQISKDLNTGFREHAEKSEM----
NCU05204.1	VHSNSNSDDAETSMISKLKEACGFEYTNKLQRMFQDMQISKDLNTGFKEHVASLNMD---
FG09616.1 	VNSNSSSDDAETSMISKLKEACGFEYTNKLQRMFQDMQISGDLNKEFRVHLEGVES----
AN1019.1  	VHVSSVSDDAETSMISKLKEACGFEYTNKLQRMFQDIQISKDLNSSYKDWLEKSFLDDDD
          	*: .* ******************************:*** ***. ::        ....

MG07145.1 	--KVLDSQYAILGTGFWPLQVPNTAFNPPAVISEDCERFTRFYKNKHDGRKLTWLWQLCK
NCU05204.1	-GKPLDSTYSILGTGFWPLVPPNTSFVAPAEISADCDRFTRFYKNKHEGRKLTWLWQLCK
FG09616.1 	-TKVVDSTFSILGTGFWPLTAPSTDFNPPPEIAAEIERFTRFYKHKHDGRKLTWMWNLCK
AN1019.1  	RKKLVDSHFQILGTGFWPLTAPSTSFLAPPEIVKTSERFQKFYCDKHNGRKLTWLWQLCK
          	  * :** : *********  *.* * .*. *    :** :** .**:******:*:***

MG07145.1 	GEVRATYCKQSKVPYTFQVSMYQMAILLMFNDGDRHTHEEIASTTSMNSETMDPVLGLLL
NCU05204.1	GDIKANYMKGAKMPYIFSVSAYQMAILLLFNEKDQYTFEELASITQLNADVLEGALGILV
FG09616.1 	GEIKASYCKASKTPYTFQVSIYQMAILLLFNEKDSYSYDDILSTTQLSKEVSDQAIAVLL
AN1019.1  	GELKANYIKNTKVPYTFLVSTYQMGILLLFNESDTLTYSDIQKATTLTPEILDPNLSIFL
          	*:::*.* * :* ** * ** ***.***:**: *  :..:: . * :. :  :  :.:::

MG07145.1 	KAKVLLAD---SDKPGPGTTYSLNYDFKNKKVRVNLNIGLKTEQKQEEVETNKTIEEDRK
NCU05204.1	KAKVLTAEGGEGGKIGPGATFSLNYDFKNKKYRINLNVGMKSETKQEEAETNKTIEEDRK
FG09616.1 	KAKVLIMSGAAGEKPGSGKTFKLNYDFKSKKIRINLNIGGVKEAKQEEVETNKTIEEDRK
AN1019.1  	KAKVLNIS-PEGSKPGPDSTFSLNYNFKNKKIKVNLNIQIRSEQKVETDDTHKTIEEDRK
          	*****  .   . * *.. *:.***:**.** ::***:   .* * *  :*:********

MG07145.1 	LLLQ0SAIVRIMKARKRMKHNQLVSETIGQIKGRFVPKIPDIKKCIEILLDKEYLERLED
NCU05204.1	LLLQ0SAIVRIMKARKKMKHQQLVSETINQIKARFMPKIGDIKKCIEILLDKEYLERLED
FG09616.1 	LVLQ0SAIVRIMKARKKMKHTQLVSETINQIRSRFVPKVGDIKKCIEILLDKEYLERLED
AN1019.1  	LLLQ0SAIVRIMKSRKKMKHVQLVQEVIHQVKSRFPPQVPDIKKNIEALMEKDYIERLDG
          	*:** ********:**:*** ***.*.* *::.** *:: **** ** *::*:*:***:.

MG07145.1 	DDLGYLA
NCU05204.1	DELGYLA
FG09616.1 	DELGYLA
AN1019.1  	DEIAYIA
          	*::.*:*
```
